# Association Between Herpes Zoster Vaccination and Dementia Risk: A Systematic Review and Meta-Analysis

**DOI:** 10.3390/vaccines14080685

**Published:** 2026-08-10

**Authors:** Qing Zhang, Quanman Hu, Yaming Wei, Jun Li, Shuaiyin Chen

**Affiliations:** 1 College of Public Health, Zhengzhou University, 100 Kexue Avenue, Zhengzhou 450001, China; 2 Huaiyang District Center for Disease Control and Prevention, Zhoukou 466700, China; 3 Henan Provincial Center for Disease Control and Prevention, Zhengzhou 450016, China

**Keywords:** dementia, herpes zoster vaccination, older adults, recombinant zoster vaccine, meta-analysis

## Abstract

Background/Objectives: Dementia represents a major global health challenge, and preventive strategies remain limited. Observational studies have suggested a possible association between herpes zoster vaccination (HZV) and dementia risk, although the influence of vaccine type and dose regimen remains unclear. Methods: PubMed, Embase, and Web of Science were searched through January 2026. The primary random-effects meta-analysis included one adjusted estimate per independent study or data-source cluster. Quasi-experimental and correlated secondary estimates were reported separately, and alternative-estimate and leave-one-out analyses retained independent statistical units. The protocol was registered in PROSPERO (CRD420261326042). Results: Fourteen reports representing 13 studies were included. HZV was associated with a lower dementia risk in the primary analysis of five independent data sources (ratio estimate = 0.72; 95% *CI*: 0.65–0.81; *I*^2^ = 97.1%), with similar sensitivity results (0.70–0.76). Two regression-discontinuity studies reported eligibility-associated absolute reductions in dementia diagnosis of 1.3 and 1.8 percentage points. Two direct vaccine comparisons favored the recombinant zoster vaccine (RZV; Shingrix) over the live attenuated vaccine (ZVL; Zostavax) (RMTL ratio = 0.83; 95% *CI*: 0.80–0.87; *RR* = 0.82; 95% *CI*: 0.69–0.98). The only direct dose comparison favored two or more RZV doses over one dose (*RR* = 0.81; 95% *CI*: 0.74–0.88). Active-comparator associations were generally attenuated, whereas age- and subtype-specific findings varied. These secondary findings were exploratory. Conclusions: HZV was associated with lower dementia risk in observational studies, with directionally consistent quasi-experimental findings. Direct evidence suggested potentially stronger inverse associations for RZV and complete vaccination, but was based on few independent data sources. Substantial heterogeneity and residual confounding preclude causal interpretation, and further prospective or randomized evidence is needed.

## 1. Introduction

Dementia, including Alzheimer’s disease, vascular dementia, and other neurodegenerative disorders, represents one of the most pressing public health challenges worldwide. By 2050, an estimated 150 million individuals will have dementia due to the ageing of the world’s population [1]. Patients, families, healthcare institutions, and society as a whole bear a high burden of illness and financial expenses due to dementia [2]. Finding modifiable risk factors and possible preventative measures is, therefore, a top public health priority. Finding workable primary preventive strategies is crucial, as there are currently no therapeutic medicines that can reverse the progression of the disease. The role that infection and persistent inflammation play in the pathogenesis of dementia has attracted considerable interest lately [3]. After a first infection, the varicella-zoster virus (VZV) lies latent in the dorsal root ganglia and can reactivate to produce shingles [4]. According to research, VZV infection may aggravate vascular damage, cause neuroinflammation, or directly invade the central nervous system to accelerate the onset and progression of dementia [5].

The biological link between immunization and shingles prevention has generated significant scientific interest in the vaccine’s potential association with dementia risk. Herpes zoster vaccine (HZV) may be associated with a lower incidence of dementia, according to preliminary results from several observational studies [6]. Important clinical questions, such as whether associations differ by herpes zoster vaccine type and whether dose regimen is associated with dementia risk, remain unresolved due to substantial differences in the available data. First, there are now two widely used vaccination types with different immunological mechanisms: recombinant subunit vaccines and conventional live attenuated vaccines [7]. It is unknown whether the two vaccines differ in their association with dementia risk, although they differ in formulation, immunogenicity, dosing schedule, and safety profiles in older adults. Additionally, a key question in clinical practice is whether receiving two doses of recombinant subunit vaccine is associated with lower dementia risk than receiving only one dose during the course of immunization. Furthermore, a systematic review is necessary, as the association between immunization and dementia risk may vary by dementia type, follow-up period, age, and sex. It should be noted that, while systematic reviews have been published in this area, none of these studies examined the crucial element of vaccination dose or distinguished between different types of vaccines [8]. As a result, it remains unclear whether associations differ by vaccine type and whether completion of the vaccination schedule is associated with dementia risk. Because of this restriction, the available data are insufficient to fully inform clinical and public health decisions on “which vaccine to administer” and “how to administer it.”

Therefore, to better understand the association between herpes zoster vaccination and dementia risk and to improve precision in dementia risk assessment, this study aims to synthesize findings from multiple studies on the association strength of different vaccine types and dosage regimens. This synthesis will provide an updated and clinically relevant assessment of the link between herpes zoster vaccination and dementia risk. The main goal of this study is to quantitatively evaluate the overall association between herpes zoster vaccination and the risk of dementia. More importantly, this study extends previous reviews by examining vaccine type, dose regimen, and dementia subtype through predefined subgroup analyses. These analyses explore potential differences in the association between dementia risk and live attenuated vaccines or recombinant subunit vaccines. Secondly, it assesses whether receiving two doses of the recombinant subunit vaccine is associated with lower dementia risk than receiving a single dose. Finally, it analyses variation in this association across different age groups and dementia subtypes and evaluates whether control group design may influence the observed association, particularly in relation to healthy-user bias. We expect this study’s findings to provide clinicians and public health policymakers with a clearer understanding of the current observational evidence on herpes zoster vaccination and dementia risk.

## 2. Materials and Methods

This systematic review and meta-analysis was conducted and reported in accordance with the Preferred Reporting Items for Systematic Reviews and Meta-Analyses (PRISMA) 2020 statement [9] and was registered with PROSPERO (registration number: CRD420261326042). The registration record is publicly accessible through PROSPERO, and no amendments were made to the registered protocol.

### 2.1. Search Strategy and Selection Criteria

#### 2.1.1. Search Strategy

This study systematically searched PubMed, EMBASE, and Web of Science databases from their inception to January 2026. The final searches were conducted on 15 January 2026 for EMBASE and on 29 January 2026 for PubMed and Web of Science. The search did not impose language restrictions. The retrieval strategy combined free-text terms with Medical Subject Headings (MeSH), and the core search terms included “herpes zoster,” “shingles,” “varicella-zoster virus,” “herpes zoster vaccine,” “dementia,” “vascular dementia,” and “Alzheimer’s disease.” Additionally, the reference lists of included studies and relevant systematic reviews were manually checked for potential additional studies. No additional eligible records were identified through this process. The complete database-specific search strategies are provided in Supplementary Table S1.

#### 2.1.2. Inclusion Criteria

Studies were eligible if they: (1) used a cohort, case–control, target-trial emulation, natural-experiment, or quasi-experimental design, including regression-discontinuity designs; (2) evaluated herpes zoster vaccination as the exposure or intervention; (3) included an unvaccinated, routine-care, active-vaccine, alternative-vaccine, or eligibility-threshold comparator; (4) evaluated incident all-cause dementia or a prespecified dementia subtype; and (5) reported an adjusted relative effect estimate, absolute risk difference, restricted mean time lost measure, or another effect measure with sufficient uncertainty information for quantitative or structured synthesis.

#### 2.1.3. Exclusion Criteria

The following types of studies were excluded: (1) conference abstracts that did not provide sufficient information on the population, exposure, comparator, outcome, study design, or effect estimate; (2) reports that duplicated an included full publication without providing additional study information; linked preliminary and full reports were treated as reports of the same underlying study and were not counted as independent effect estimates; (3) cross-sectional studies; (4) studies evaluating cognitive impairment, dementia progression, or dementia-related mortality without reporting incident dementia; and (5) studies without an extractable effect estimate and corresponding measure of uncertainty.

### 2.2. Research Selection

Using eligibility criteria and exclusion standards, two reviewers independently assessed the literature included in this study. First, duplicate publications were eliminated, along with those deemed irrelevant to the study based on their titles and abstracts. Subsequently, potential articles were identified, downloaded, and thoroughly examined to ensure data could be integrated appropriately. When there was a debate about whether to include an article, the two researchers consulted a third reviewer to reach a decision.

### 2.3. Data Extraction

The first author, publication year, country, study type, study design, sample size, participant age, participant gender, vaccine type, definition of the control group, dementia outcome, and primary adjusted confounders (briefly listed) were independently retrieved and summarized by two authors from each included study. A third author verified the extracted data. For studies reporting multiple effect estimates, we recorded the population, vaccine type, comparator group, follow-up period, outcome definition, and whether the estimate was eligible for the non-overlapping primary analysis or retained for alternative-estimate sensitivity analyses or secondary descriptive analyses. Potential overlap was assessed using the country, database, study period, eligibility criteria, author group, and exposure and comparator definitions; reports with overlapping or potentially overlapping populations were assigned to the same data-source cluster. One RZV dose was classified as partial and two or more doses as complete vaccination, whereas ZVL was generally treated as a single-dose exposure. Dementia outcomes were classified as all-cause dementia, Alzheimer’s disease, vascular dementia, or other dementia, with administrative-code ascertainment recorded separately.

### 2.4. Quality Assessment

The Newcastle–Ottawa Scale (NOS) was used to assess the quality of non-randomized studies. The NOS is a method for grading research that assigns scores based on the extent to which researchers meet objectives across three distinct domains: selection, comparability, and outcome [10]. Two reviewers independently assessed the risk of bias of each included study using the NOS. Disagreements were resolved through discussion and, when necessary, consultation with a third reviewer. Studies scoring 7–9 were considered high quality, those scoring 4–6 moderate quality, and those scoring 0–3 low quality. Because the NOS does not specifically capture healthy-vaccine bias, healthcare-utilization bias, protopathic bias, or all design-specific assumptions of quasi-experimental studies, these threats to validity were additionally evaluated narratively on the basis of comparator design, covariate adjustment, exposure timing, and outcome ascertainment. High NOS scores were therefore not interpreted as excluding these biases.

### 2.5. Statistical Analysis

We extracted adjusted hazard ratios (HRs), risk ratios (RRs), odds ratios (ORs), absolute risk differences (RDs), restricted mean time lost (RMTL) ratios, and their 95% confidence intervals (CIs). HRs, RRs, and ORs from broadly comparable conventional studies were analysed on the log scale and reported as pooled ratio estimates; they were not assumed to represent mathematically identical causal estimands. The primary analysis included one estimate per independent study population or data-source cluster. When multiple estimates were available, we prioritized the estimate with the longest follow-up, the most complete vaccination definition, and the most complete adjustment or author-reported combined result. Alternative estimates replaced rather than supplemented the corresponding primary estimates. Regression-discontinuity eligibility effects were reported individually on the absolute RD scale and were not pooled because only two studies were available. Direct RZV-versus-ZVL estimates were also reported individually and were not pooled because the studies used different effect measures and estimands.

Random-effects models using the DerSimonian–Laird estimator and Hartung–Knapp-adjusted CIs were applied to the primary, exploratory ZVL, and sensitivity meta-analyses. Heterogeneity was assessed using the Cochrane Q test, I^2^ statistic, and τ^2^ [11,12]. Four independent conventional ZVL or predominantly ZVL estimates were pooled, whereas the single independent conventional RZV estimate was displayed separately without a formal between-platform test. Correlated estimates relating to dose regimen, comparator design, age, and dementia subtype were presented descriptively without pooled subgroup estimates or formal between-group tests. Alternative-estimate analyses retained five independent units, and leave-one-out analysis was restricted to the five primary estimates. Funnel plots and Egger’s regression test were not performed because fewer than 10 independent estimates were available. Analyses were conducted using R version 4.4.3. No formal assessment of evidence certainty, such as GRADE, was conducted. Multilevel and robust-variance models were considered but were not used because too few independent data-source clusters were available within the secondary analyses for reliable variance estimation. Correlated estimates were therefore displayed descriptively or replaced by one representative independent estimate.

## 3. Results

### 3.1. Research Summary

**Figure 1:**
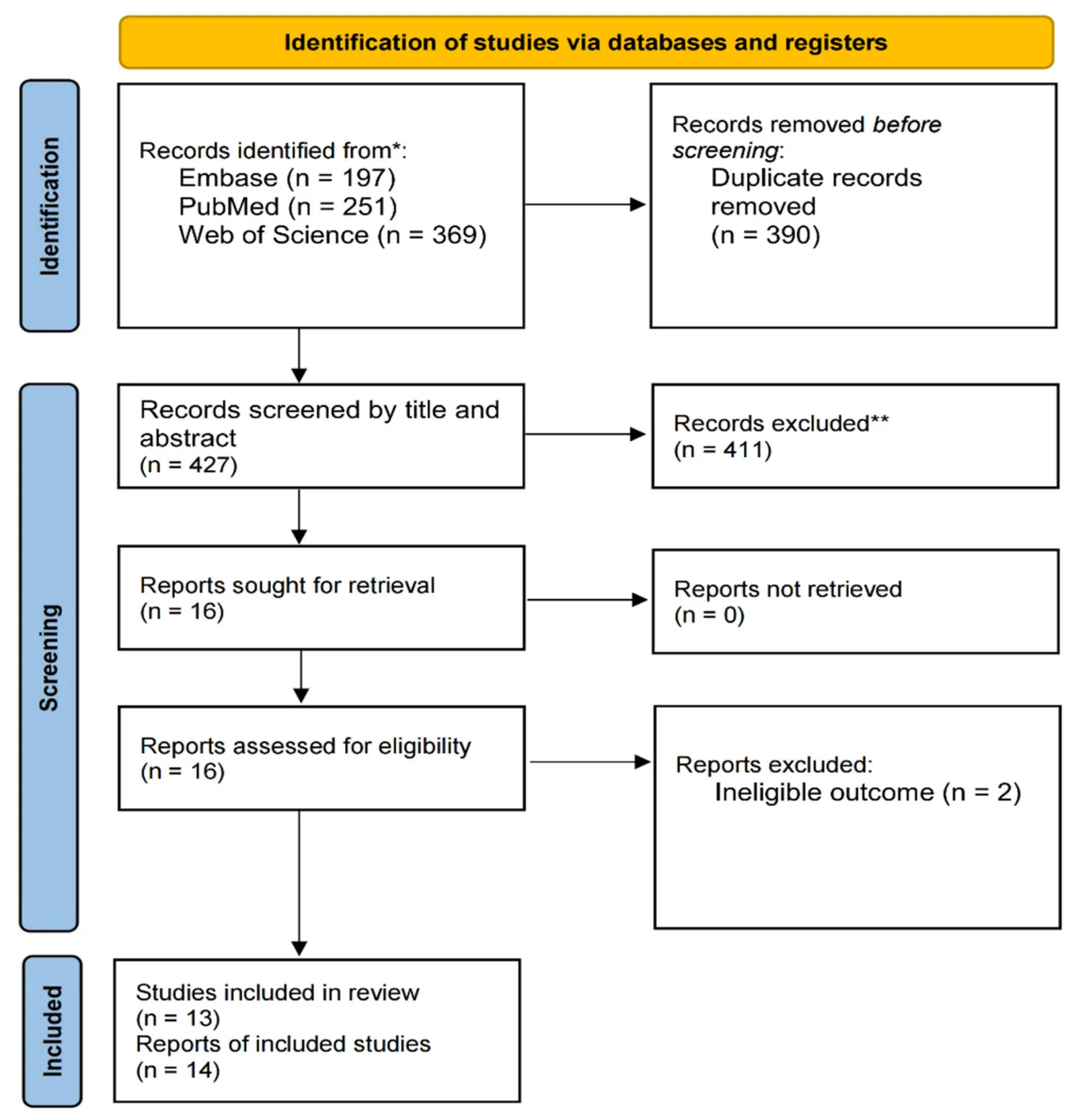
PRISMA 2020 flow diagram of study selection. The searches identified 817 records, of which 427 remained after duplicate removal. Sixteen reports underwent full-text assessment; two were excluded because they did not evaluate the eligible incident-dementia outcome. Fourteen reports representing 13 studies were included in the systematic review. * Records were identified from Embase, PubMed, and Web of Science. ** All title-and-abstract exclusions were made manually; no automation tools were used. Abbreviations: PRISMA, Preferred Reporting Items for Systematic Reviews and Meta-Analyses.

The database searches identified 817 records: 251 from PubMed, 197 from Embase, and 369 from Web of Science. After removal of 390 duplicate records, 427 records were screened by title and abstract, and 16 reports were assessed in full text. Two reports were excluded because they did not evaluate incident dementia as the eligible outcome. Fourteen reports describing 13 studies were included in the systematic review. The number of independent studies contributing to each quantitative synthesis varied according to the data source, exposure contrast, outcome, and availability of compatible effect measures (Figure 1).

Fourteen reports representing 13 studies, published between 2021 and 2025, were included from the United States, the United Kingdom (including Wales), and Australia. Study designs included retrospective cohorts, nested case–control and matched-cohort analyses, and natural or quasi-experimental studies using vaccine-policy or date-of-birth eligibility thresholds (Supplementary Table S2). Vaccine exposures included ZVL, RZV, combined or unspecified HZV, and direct RZV-versus-ZVL comparisons; comparators included unvaccinated or routine-care populations, active or alternative vaccines, and eligibility thresholds. Tang et al. [13] reported partial and complete RZV estimates, whereas Polisky et al. [14] provided the only direct comparison of two or more RZV doses versus one dose. Harris et al. [15] was retained descriptively because of potential overlap with other Optum datasets, and Schwab et al. [16] was treated as a linked preliminary report of the Polisky study rather than an independent study or estimate.

Several reports contributed correlated estimates or used overlapping electronic health-record or administrative data sources. Aggregate sample sizes are therefore reported as participant records and should not be interpreted as unique individuals. Supplementary Table S3 maps data-source overlap and the handling of correlated estimates, whereas Supplementary Table S4 lists each estimate and its analysis-specific role. Each quantitative synthesis retained only one estimate per independent study population or data-source cluster. All 13 studies scored 7–9 on the NOS, although these scores do not exclude healthy-vaccine, healthcare-utilization, protopathic, outcome-misclassification, or design-specific biases.

### 3.2. Quantitative Synthesis

#### 3.2.1. Overall Effect Analysis

**Figure 2:**
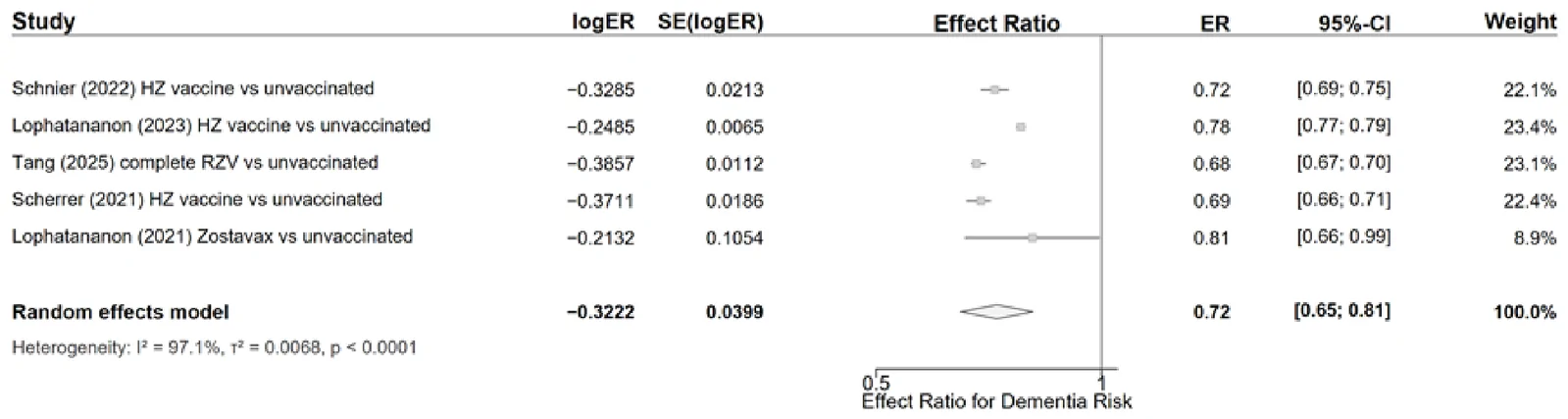
Forest plot of the primary study-level analysis of herpes zoster vaccination and all-cause dementia risk [13,17,18,19,20]. The primary analysis was restricted to one estimate per independent study population or data-source cluster to reduce double-counting of participants and correlated estimates. The pooled ratio estimate and 95% confidence interval were calculated using a random-effects model with Hartung–Knapp adjustment. Squares represent individual study estimates, with square size proportional to study weight; horizontal lines represent 95% CIs; the diamond represents the pooled estimate. Abbreviations: CI, confidence interval; SE, standard error.

The primary analysis included five study-level estimates selected to minimize overlap across populations and data sources (Figure 2) [13,17,18,19,20]. HZV was associated with a lower risk of all-cause dementia, with a pooled ratio estimate of 0.72 (95% *CI*: 0.65–0.81). Heterogeneity remained substantial (*I*^2^ = 97.1%, *τ*^2^ = 0.0068, *p* < 0.0001). These findings indicate an inverse summary association, although the substantial heterogeneity and observational design limit causal interpretation.

#### 3.2.2. Evidence from Regression-Discontinuity Studies

Two independent studies used date-of-birth eligibility thresholds to evaluate the association between ZVL eligibility and incident dementia. Eyting et al. [21] reported that eligibility for ZVL was associated with a 1.3-percentage-point reduction in the probability of a new dementia diagnosis over 7 years (95% *CI*: 0.2–2.7). Pomirchy et al. [22] reported an eligibility-associated reduction of 1.8 percentage points over 7.4 years (95% *CI*: 0.4–3.3). These estimates were reported individually on the absolute risk-difference scale and were not pooled because only two studies with differing populations, vaccination programmes, and follow-up periods were available. Both estimates were directionally consistent with the primary association.

#### 3.2.3. Vaccine-Type Evidence

**Figure 3:**
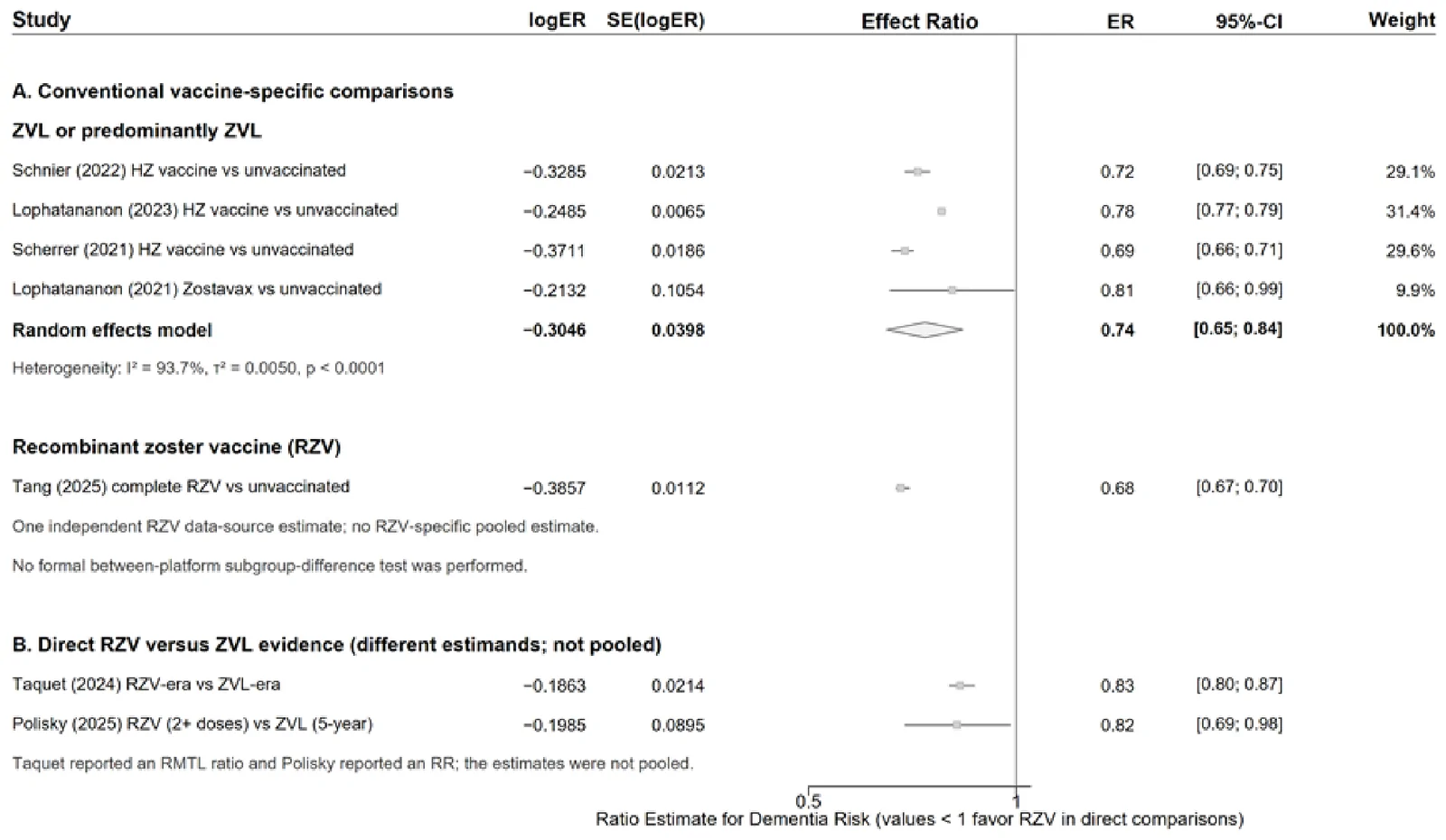
Forest plot of evidence according to vaccine platform. Panel (**A**) presents four independent conventional estimates involving ZVL or predominantly ZVL exposure and their exploratory random-effects summary, together with the single independent conventional RZV estimate [13,17,18,19,20]. No formal between-platform subgroup test was performed. Panel (**B**) presents two direct RZV-versus-ZVL estimates. These estimates were displayed separately and were not pooled because Polisky et al. [14] reported an RR, whereas Taquet et al. [23] reported an RMTL ratio. Square markers represent individual estimates; the diamond represents the pooled ZVL summary. Values below 1 favour vaccination or RZV according to the comparison shown. Abbreviations: CI, confidence interval; ER, effect ratio; RR, risk ratio; RZV, recombinant zoster vaccine; ZVL, live zoster vaccine; RMTL, restricted mean time lost.

We performed an exploratory subgroup analysis to examine whether the association differed by vaccine type (Figure 3A). Four independent estimates evaluated ZVL or predominantly ZVL vaccination. Their exploratory random-effects synthesis yielded a pooled ratio estimate of 0.74 (95% *CI*: 0.65–0.84), with substantial heterogeneity (*I*^2^ = 93.7%). Only one independent RZV estimate met the same analysis criteria: Tang et al. [13] reported an HR of 0.68 (95% *CI*: 0.67–0.70) for complete RZV vaccination versus no RZV vaccination. Because only one independent conventional RZV estimate was available, no formal between-platform subgroup test was performed. Direct head-to-head evidence was therefore considered separately.

Direct RZV-versus-ZVL evidence was available from two independent studies (Figure 3B). Taquet et al. [23] used the rapid transition from ZVL to RZV as a natural experiment and reported an RMTL ratio of 0.83 (95% *CI*: 0.80–0.87). Polisky et al. [14] reported an RR of 0.82 (95% *CI*: 0.69–0.98) for two-or-more-dose RZV versus ZVL. Schwab et al. [16] was a linked preliminary report of the Polisky study and was therefore not treated as an additional independent estimate. The Taquet and Polisky estimates were presented separately because they represented different effect measures and estimands. Both estimates were below 1 and directionally favoured RZV; however, the evidence was derived from only two independent data sources and does not establish platform superiority. These findings should therefore be considered hypothesis-generating, and additional independent head-to-head studies are needed.

#### 3.2.4. Dose-Regimen Evidence

**Figure 4:**
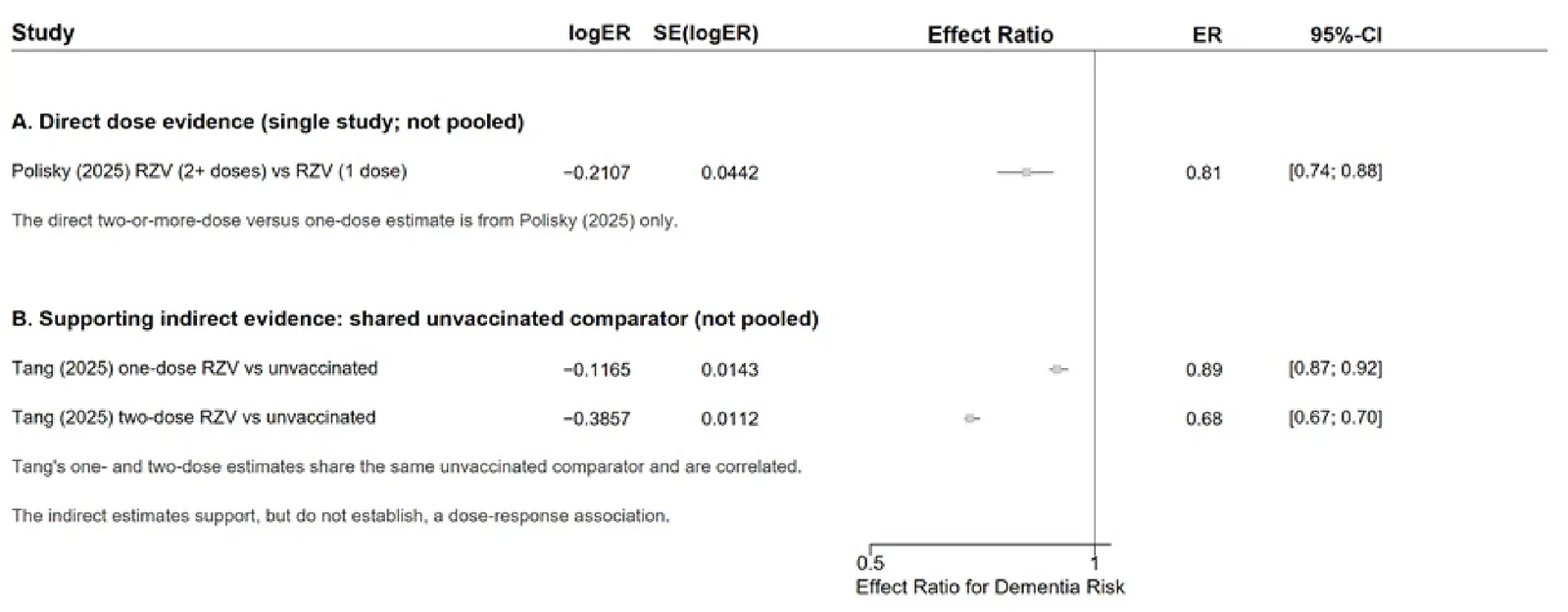
Forest plot of direct and indirect evidence regarding RZV dose regimen. Evidence from Tang et al. [13] and Polisky et al. [14] is shown. Panel (**A**) presents the direct comparison of two or more RZV doses versus one dose reported by Polisky et al. Panel (**B**) presents the correlated one-dose and two-dose estimates reported by Tang et al., each compared with the same unvaccinated population. The indirect estimates were displayed separately without pooling or a formal between-dose test because they shared the same comparator population. Square markers represent individual estimates. Abbreviations: CI, confidence interval; ER, effect ratio; RZV, recombinant zoster vaccine.

We examined whether completion of the two-dose RZV schedule was associated with a lower risk of dementia (Figure 4). The most directly relevant evidence was provided by Polisky et al. [14], who compared recipients of two or more RZV doses with recipients of one dose within the same study framework. Receipt of two or more doses was associated with a lower observed dementia risk than receipt of one dose (*RR* = 0.81, 95% *CI*: 0.74–0.88), corresponding to a 19% lower observed relative risk. Although this direct estimate was derived from a single study, it provides preliminary direct evidence of a potentially stronger inverse association following completion of the RZV schedule. Differences in vaccination adherence and health-seeking behaviour may nevertheless have influenced the observed association.

Supporting indirect evidence was provided by Tang et al. [13], who reported an HR of 0.89 (95% *CI*: 0.87–0.92) for one-dose RZV and an HR of 0.68 (95% *CI*: 0.67–0.70) for two-dose RZV, each compared with the same unvaccinated population. The more pronounced inverse association observed for two-dose vaccination was directionally consistent with the direct comparison reported by Polisky et al. [14]. However, because the Tang estimates shared the same comparator population, they were correlated and were therefore displayed separately without formal pooling or a between-dose statistical test. Taken together, the concordant direct and indirect findings provide preliminary support for a potentially stronger inverse association following completion of the two-dose RZV schedule. Given that the direct comparison was derived from one study, this finding should be considered hypothesis-generating and warrants confirmation in additional independent studies.

#### 3.2.5. Comparator-Design Analysis

We examined estimates obtained using different comparator designs to assess the potential sensitivity of the observed association to healthy-vaccine bias (Supplementary Figure S1). The five independent comparisons using unvaccinated or routine-care controls corresponded to the primary analysis and yielded a pooled ratio estimate of 0.72 (95% *CI*: 0.65–0.81). In the Polisky study, the active-comparator estimates were 0.73 (95% *CI*: 0.70–0.75) for ZVL versus PPSV23 and 0.83 (95% *CI*: 0.75–0.92) for two-or-more-dose RZV versus PPSV23. These two estimates shared the same PPSV23 comparator population and were therefore displayed separately without statistical pooling. Taquet et al. [24] separately reported an RMTL ratio of 0.82 (95% *CI*: 0.74–0.91) for RZV versus influenza vaccination.

All active-comparator estimates remained below 1, suggesting that the inverse association was not confined to comparisons with unvaccinated populations. The active-comparator point estimates, particularly those involving RZV, were generally closer to the null than several estimates obtained using unvaccinated comparators. However, these estimates were not directly comparable because the studies differed in vaccine type, population, data source, effect measure, and comparator definition. Consequently, no pooled active-comparator estimate or formal between-comparator test was calculated. Taken together, the active-comparator findings remain compatible with an inverse association while also suggesting that healthy-vaccine bias may partly contribute to its observed magnitude.

#### 3.2.6. Age-Specific Evidence

Age-specific estimates were examined descriptively by data source because multiple correlated age-stratified estimates were reported within the same cohorts and the available age categories were not fully comparable across studies (Supplementary Figure S2). In the VHA cohort reported by Scherrer et al. [19], the ratio estimates were 0.61 (95% *CI*: 0.54–0.68) for participants aged 65–69 years, 0.83 (95% *CI*: 0.76–0.91) for those aged 70–74 years, and 0.66 (95% *CI*: 0.63–0.68) for those aged 75 years or older. In the MarketScan cohort, the corresponding estimates were 1.00 (95% *CI*: 0.79–1.27), 0.65 (95% *CI*: 0.52–0.81), and 0.59 (95% *CI*: 0.50–0.69), respectively. Polisky et al. [14] separately reported an estimate of 0.73 (95% *CI*: 0.60–0.90) among women aged 80–89 years; this narrower, sex-specific subgroup was not directly comparable with the broader age categories reported by Scherrer et al. [19].

Age-specific patterns were not consistent across data sources. The MarketScan point estimates suggested a progressively stronger inverse association in older age groups, whereas the VHA estimates did not show a monotonic age-related pattern. Because the estimates were correlated within cohorts, the age categories differed across studies, and only a limited number of independent data sources were available, no pooled age-specific estimates or formal between-age subgroup tests were calculated.

#### 3.2.7. Dementia-Subtype Evidence

Dementia-subtype estimates were presented descriptively by study and data source because multiple outcomes reported within the same study were correlated and some reports used potentially overlapping databases (Supplementary Figure S3). Schnier et al. [17] reported ratio estimates of 0.81 (95% *CI*: 0.77–0.86) for Alzheimer’s disease and 0.66 (95% *CI*: 0.61–0.71) for vascular dementia. Lophatananon et al. [20] reported estimates of 0.91 (95% *CI*: 0.89–0.92) for Alzheimer’s disease and 0.71 (95% *CI*: 0.69–0.72) for other dementia, whereas Douros et al. [25] reported an estimate of 1.01 (95% *CI*: 0.97–1.05) for Alzheimer’s disease using a potentially overlapping CPRD population. In the Polisky study [14], the active-comparator estimates were 0.51 (95% *CI*: 0.45–0.58) for Alzheimer’s disease and 0.42 (95% *CI*: 0.36–0.50) for vascular dementia.

In both studies that reported Alzheimer’s disease and vascular dementia within the same study framework, the point estimate was lower for vascular dementia. This within-study pattern suggests that the inverse association may be more pronounced for vascular dementia than for Alzheimer’s disease. However, because the outcome-specific estimates were correlated and only a limited number of independent data sources were available, no pooled subtype-specific estimates or formal between-subtype tests were calculated. The observed subtype pattern should therefore be considered hypothesis-generating and warrants confirmation in additional independent studies.

### 3.3. Sensitivity Analysis and Leave-One-Out Analysis

#### 3.3.1. Sensitivity Analysis

To assess whether the primary result was sensitive to the selection of a representative estimate from the same or potentially overlapping data-source cluster, we performed three alternative-estimate sensitivity analyses (Supplementary Figure S4). In each scenario, one primary estimate was replaced, rather than supplemented, by an alternative eligible estimate, and the number of independent units remained five. Replacing Scherrer et al. [19] with Wiemken et al. [26] yielded a pooled ratio estimate of 0.74 (95% *CI*: 0.66–0.82; *I*^2^ = 96.6%). Replacing Lophatananon et al. (2023) [20] with Douros et al. [25] yielded a pooled ratio estimate of 0.76 (95% *CI*: 0.62–0.94; *I*^2^ = 98.7%). Replacing Tang et al. [13] with the Optum-based RZV active-comparator estimate from Polisky et al. [14] yielded a pooled ratio estimate of 0.75 (95% *CI*: 0.68–0.83; *I*^2^ = 92.0%). All alternative pooled estimates remained below 1.00, with confidence intervals excluding the null value. These analyses support the robustness of the direction of the primary association to alternative estimate selection, although substantial heterogeneity persisted.

#### 3.3.2. Leave-One-Out Analysis

The leave-one-out analysis was restricted to the five independent data-source estimates included in the primary model (Supplementary Figure S5). Four independent estimates remained in each iteration. Sequential omission of Schnier et al. [17], Lophatananon et al. (2023) [20], Tang et al. [13], Scherrer et al. [19], and Lophatananon et al. (2021) [18] yielded pooled ratio estimates ranging from 0.70 to 0.74, with all corresponding 95% confidence intervals remaining below 1.00. No single omission reversed the direction of the pooled association or materially altered its magnitude.

## 4. Discussion

This systematic review and meta-analysis examined the association between HZV and dementia risk using data from 14 reports representing 13 studies, collectively encompassing more than 15 million participant records. Because several reports used overlapping databases or linked populations, this number should not be interpreted as representing unique individuals. In the primary analysis restricted to five independent study- or data-source estimates, HZV was associated with a lower risk of all-cause dementia (pooled ratio estimate = 0.72, 95% *CI*: 0.65–0.81). Alternative-estimate sensitivity analyses produced similar pooled estimates ranging from 0.74 to 0.76, while leave-one-out estimates ranged from 0.70 to 0.74. Two regression-discontinuity studies also reported eligibility-associated reductions in dementia diagnosis of 1.3 and 1.8 percentage points, respectively. These estimates were reported individually and were not statistically pooled because only two studies were available and they differed in population, vaccination programme, healthcare setting, and follow-up duration. However, heterogeneity remained substantial across analyses, indicating that differences in vaccine type, dose regimen, comparator design, dementia definition, population characteristics, and data sources may have contributed to variation in the observed estimates. Therefore, the secondary findings should be interpreted as exploratory rather than as definitive evidence of effect modification.

The subgroup findings suggest that vaccine-specific factors may contribute to the observed heterogeneity. Among conventional cohort estimates, four independent studies involving ZVL or predominantly ZVL exposure yielded a pooled ratio estimate of 0.74, whereas only one independent conventional RZV estimate was available. Therefore, no formal between-platform subgroup comparison was performed. Two direct RZV-versus-ZVL comparisons reported estimates favouring RZV (RMTL ratio = 0.83 and *RR* = 0.82), but these estimates were not pooled because they represented different effect measures. Although these findings suggest a possible advantage of RZV, they were derived from only two independent data sources and do not establish platform superiority. RZV is an adjuvanted recombinant vaccine administered as a two-dose regimen and has shown strong efficacy against herpes zoster in older adults, while also inducing robust cellular immune responses [27,28]. These characteristics provide biological plausibility for a possible difference between vaccine types, but the present evidence cannot determine whether this mechanism explains the observed association with dementia risk. Dose-regimen findings also suggested a stronger association among individuals receiving two or more RZV doses than among those receiving one dose (*RR* = 0.81) [14]. Nevertheless, this was the only direct dose comparison, and completion of vaccination may also correlate with health-seeking behaviour. Therefore, the vaccine-type and dose findings should be considered hypothesis-generating.

The dementia-subtype analysis also suggested possible variation across outcome definitions. In studies reporting both AD and VaD, the point estimates for VaD were lower than the corresponding estimates for AD. However, these outcomes were derived from the same study populations and therefore represented correlated rather than independent estimates. This pattern is biologically plausible because VZV reactivation has been linked to vascular inflammation, vasculopathy, and stroke-related mechanisms, which may be more relevant to VaD than to AD pathology [29]. Nevertheless, dementia-subtype classification relied largely on diagnostic coding, and the available estimates varied across studies. Age-specific estimates also varied across databases, providing insufficient evidence to establish effect modification by age. Therefore, the dementia-subtype and age findings should be considered hypothesis-generating rather than conclusive evidence of differential associations.

Comparator design is an important methodological issue in the literature. We examined comparator design and active-comparator evidence to explore the potential influence of healthy-vaccine bias [30]. Active-comparator estimates remained below 1, although some were attenuated relative to estimates using unvaccinated or routine-care controls. Because some active-comparator estimates shared a comparator group or used different effect measures, they were presented separately rather than pooled, and no formal between-group test was performed. This attenuation is compatible with the possibility that differences in health-seeking behaviour and baseline health status partly contribute to the observed association. However, the persistence of inverse associations with active comparators remains compatible with an association while neither proving causality nor eliminating residual confounding.

Several limitations should be considered. First, all available evidence was nonrandomized, including the natural-experiment and regression-discontinuity studies; residual confounding, healthy-vaccine bias, healthcare-utilization bias, and protopathic bias therefore remain possible. Healthy-vaccine and healthcare-utilization biases may exaggerate the inverse association, whereas coding-based outcome misclassification may attenuate or obscure subtype-specific associations. Second, overlapping databases and correlated estimates were addressed by selecting one estimate per independent data-source cluster and using replacement sensitivity analyses, although undocumented overlap cannot be excluded. Third, HRs, RRs, ORs, and RMTL ratios represent different estimands, and incompatible measures were therefore reported separately. Substantial heterogeneity also indicates that pooled results represent average associations across diverse settings rather than a uniform effect. Finally, platform-, dose-, age-, and subtype-specific evidence was sparse, and publication-bias tests were not performed because the primary analysis included fewer than 10 independent estimates. The current evidence is insufficient to recommend HZV specifically for dementia prevention; vaccination decisions should continue to follow established indications for herpes zoster prevention.

## 5. Conclusions

In summary, herpes zoster vaccination was associated with a lower risk of dementia in observational studies, with directionally consistent findings from quasi-experimental studies. Direct comparisons suggested potentially stronger inverse associations for RZV than ZVL and for two or more RZV doses than one dose, but these findings were based on few independent data sources and should be interpreted cautiously. While these findings add to the growing observational evidence concerning the association between HZV and dementia risk, substantial heterogeneity and residual confounding limit causal interpretation, and causal relationships remain to be established through future prospective or randomized studies.

## Data Availability

All data extracted and analyzed in this study are available within the article and Supplementary Materials. Additional extracted data and the analytic code are available from the corresponding author upon reasonable request.

## Supplementary Materials

The following supporting information can be downloaded at: https://www.mdpi.com/article/10.3390/vaccines14080685/s1, Supplementary Table S1: Full search strategies for PubMed, Embase, and Web of Science; Supplementary Table S2: Basic characteristics and risk-of-bias assessments of included studies; Supplementary Table S3: Study- and data-source overlap map and handling of correlated estimates; Supplementary Table S4: Extracted effect estimates and their analysis-specific roles; Supplementary Figure S1: Forest plot of evidence according to comparator design; Supplementary Figure S2: Forest plot of age-specific estimates by data source; Supplementary Figure S3: Forest plot of study-specific estimates by dementia subtype; Supplementary Figure S4: Alternative-estimate sensitivity analyses retaining five independent data-source units; Supplementary Figure S5: Leave-one-out sensitivity analysis of the five independent primary-analysis data sources. The studies cited in the Supplementary Materials are included in the main reference list [13,14,15,16,17,18,19,20,21,22,23,24,25,26].
