# Supplementary material for: Association Between Herpes Zoster Vaccination and Dementia Risk: A Systematic Review and Meta-Analysis
Source: Vaccines (Basel). 2026 Aug 10;14(8):685. doi: 10.3390/vaccines14080685 (PMC13517738; doi:10.3390/vaccines14080685)
Supplement: Supplementary file 1 [file vaccines-14-00685-s001.zip › vaccines-4474809-supplementary.pdf]

Supplementary Information (SI)

Association Between Herpes Zoster Vaccination and Dementia Risk: A Systematic Review and Meta-Analysis

A. Supplementary tables (Table S1 to S4)

Supplementary Table S1. Full search strategies for PubMed, Embase, and Web of Science

Database and Platform	Date Last Searched	Search Strategy	Limits Applied	Records Retrieved
PubMed	29 January 2026	The complete PubMed search strategy is presented below.	Entry Date from database inception through 29 January 2026; no language restrictions	251
Embase (Embase.com)	15 January 2026	The complete Embase search strategy is presented below.	No language or document-type restrictions	197
Web of Science Core Collection	29 January 2026	The complete Web of Science search strategy is presented below.	All subscribed Web of Science Core Collection indexes; no language or document-type restrictions	369

PubMed Search Strategy

```
(
   "Dementia"[MeSH Terms]
   OR dementia[Title/Abstract]
   OR alzheimer*[Title/Abstract]
   OR "cognitive decline"[Title/Abstract]
   OR "cognitive impairment"[Title/Abstract]
   OR "cognitive dysfunction"[Title/Abstract]
   OR "risk of dementia"[Title/Abstract]
   OR "incident dementia"[Title/Abstract]
)
AND
(
   "Varicella-zoster virus reactivation"[Title/Abstract]
   OR "virus reactivation"[Title/Abstract]
   OR "viral reactivation"[Title/Abstract]
   OR "herpes zoster infection"[Title/Abstract]
   OR "Herpes Zoster"[Title/Abstract]
   OR Shingles[Title/Abstract]
   OR "Varicella Zoster Virus"[Title/Abstract]
   OR VZV[Title/Abstract]
   OR "herpes zoster vaccine"[MeSH Terms]
   OR Shingrix[Title/Abstract]
   OR RZV[Title/Abstract]
   OR Zostavax[Title/Abstract]
   OR "shingles vaccine"[Title/Abstract]
```

OR "recombinant shingles vaccine"[Title/Abstract]
OR "HZ/su"[Title/Abstract]
OR "zoster vaccination"[Title/Abstract]
)
AND
(
"1900/01/01"[Entry Date] : "2026/01/29"[Entry Date]
)

Embase Search Strategy

#1
'herpes zoster vaccine'/exp
OR 'herpes zoster vaccine'
OR 'shingles vaccine'
OR 'varicella zoster vaccine'
OR 'zoster vaccine'
OR 'zostavax'
OR 'shingrix'
OR 'live attenuated zoster vaccine'
OR 'recombinant zoster vaccine'
OR 'rzv'
OR 'lzv'
OR ((herpes NEAR/3 zoster) AND vaccin*)
OR (shingles AND vaccin*)

#2
'dementia'/exp
OR 'mild cognitive impairment'/exp
OR 'alzheimer disease'/exp
OR 'vascular dementia'/exp
OR dementia
OR 'alzheimer disease'
OR 'alzheimer dementia'
OR 'mild cognitive impairment'
OR mci
OR 'cognitive decline'
OR 'cognitive impairment'
OR 'cognitive dysfunction'
#3
#1 AND #2

Web of Science Core Collection Search Strategy

#1
TS=("herpes zoster vaccine"
OR Shingrix
OR RZV

OR Zostavax
OR "shingles vaccine"
OR "recombinant shingles vaccine"
OR HZ/su
OR "zoster vaccination")
#2
TS=("Herpes Zoster"
OR Shingles
OR "Varicella Zoster Virus"
OR VZV)
#3
TS=("Varicella-zoster virus reactivation"
OR "virus reactivation"
OR "viral reactivation"
OR "herpes zoster infection")
#4
TS=(dementia
OR alzheimer*
OR "cognitive decline"
OR "risk of dementia")
#5
#1 OR #2 OR #3
#6
#4 AND #5

Supplementary Table S2. Basic characteristics and risk-of-bias assessments of included studies

Study ID	Author (Year)	Country	Study Design	Data Source / Population	Total Sample Size (Vaccinated/ Unvaccinated)	Mean/ Starting Age	Vaccine Type	Control Group Definition	Dementia Outcome(s)	Main Adjusted Confounders (Brief)	NOS Score
1	Schnier (2022)	UK	Retrospective cohort	SAIL Database (Wales)	336,341 (155,972 / 180,369)	70 years	Mostly ZVL	Unvaccinated	All-cause, AD, VaD	Cox model adjusted for frailty, comorbidities, and SES	9
2	Wiemken (2022)	USA	Retrospective cohort	VHA & MarketScan	VHA: 135,626; MS: 129,200	VHA: 76.8; MS: 70.5	HZ (unspecified)	No HZ or Tdap vaccine	All-cause (ICD codes)	IPTW balanced demographics, comorbidities, medications, and healthcare utilization	9
3	Lophatananon (2023)	UK	Retrospective matched cohort	CPRD Aurum	~9,345,558 (854,745 / ~8,490,813)	≥70 years	ZVL (non-RZV)	Matched unvaccinated	All-cause, AD, other	Matching + adjusted for deprivation index, time varying CCI	9
4	Tang (2025)	USA	Retrospective time-dependent cohort	Optum EHR	4,502,678 (666,710 / 3,835,968)	Median 62 years	RZV	Unvaccinated for RZV	All-cause (ICD-10)	Time-dependent Cox model adjusted for age, CCI, HZ infection, etc.	7
5	Polisky (2025)[a]	USA	Retrospective matched cohort	Optum EHR	>100,000 pairs Per comparison	≥50 years	RZV & ZVL	PPSV23 vaccinated (active control)	All-cause (ICD codes)	PS matched on ~400 covariates	9
6	Scherrer (2021)	USA	Retrospective cohort (IPTW)	VHA & MarketScan	VHA: 136,016; MS: 172,790	≥65 years	HZ (mostly ZVL)	Unvaccinated	All-cause, AD	IPTW adjusted for demographics, comorbidities, medication s, and healthcare utilization	7

Study ID	Author (Year)	Country	Study Design	Data Source / Population	Total Sample Size (Vaccinated/ Unvaccinated)	Mean/ Starting Age	Vaccine Type	Control Group Definition	Dementia Outcome(s)	Main Adjusted Confounders (Brief)	NOS Score
7	Lophatananon (2021)	UK	Nested case–control	UK Biobank	Cases: 2,378; Controls: 225,845	Mean ~66–69 years	ZVL	Unvaccinated	All-cause (multiple sources)	Logistic regression adjusted for age, sex, and CCI	7
8	Douros (2023)	UK	Nested case–control	CPRD	Cases: 212,562; Controls: 846,891	Mean 70.5 years	HZ vaccine	Unvaccinated	All-cause, AD	Conditional logistic regression adjusted for extensive comorbidities, medications, and SES	9
9	Harris (2023)	USA	Retrospective cohort (PSM)	Optum Clinformatics	>100,000 pairs Per vaccine group	≥65 years	ZVL & RZV (combined)	Unvaccinated for the target vaccine	AD (ICD + medication)	PSM adjusted for demographics, comorbidities, medications, and healthcare utilization	7
10	Taquet (2024)	USA	Retrospective matched cohort	TriNetX EHR	RZV: 103,837 ZVL: 103,837	≥65 years	RZV	ZVL recipients	All-cause dementia (ICD-10)	PS matched on 60 covariates	8
11	Eyting (2025)	UK	Natural experiment (fuzzy RDD/IV)	SAIL Databank (Wales)	282,541 total (56,098 in RDD)	Mean 77.9 years	ZVL	Age-ineligible adults	All-cause dementia	Age-adjusted RDD; balance checks	9
12	Taquet (2025)	USA	Retrospective matched cohort	TriNetX EHR	RZV: 103,798 Influenza: 103,798	Mean 69.2 years	RZV	Influenza vaccine	All-cause dementia (ICD-10)	PS matched on 66 covariates	7

Study ID	Author (Year)	Country	Study Design	Data Source / Population	Total Sample Size (Vaccinated/ Unvaccinated)	Mean/ Starting Age	Vaccine Type	Control Group Definition	Dementia Outcome(s)	Main Adjusted Confounders (Brief)	NOS Score
13	Pomirchy (2025)	Australia	Quasi-experimental cohort (RDD)	PenCS primary-care EHR	Eligible: 13,743 Ineligible: 4,659	Mean 77.0 years	ZVL	Age-ineligible adults	All-cause dementia	Local linear RDD; balance checks	7

Abbreviations: AD = Alzheimer's disease; CCI = Charlson Comorbidity Index; CPRD = Clinical Practice Research Datalink; EHR = Electronic Health Records; HZ = herpes zoster; IPTW = inverse probability of treatment weighting; NOS = Newcastle–Ottawa Scale; OR = odds ratio; PS = propensity score; PSM = propensity score matching; PPSV23 = pneumococcal polysaccharide vaccine; RR = risk ratio; RZV = recombinant zoster vaccine (Shingrix); SES = socioeconomic status; VaD = vascular dementia; VHA = Veterans Health Administration; ZVL = zoster vaccine live.

a: Schwab et al. was a preliminary conference report linked to the study subsequently published in full by Polisky et al. It was counted as an additional report but not as a separate study or independent effect estimate.

Supplementary Table S3. Study- and Data-Source Overlap Map and Handling of Correlated Estimates

Study/report	Country	Data source and period	Vaccine/comparator	Outcome	Potential overlap	Shared groups/multiple estimates	Handling in analyses
Scherrer (2021)	USA	VHA, 2008–2019; MarketScan, 2009–2018	HZ vaccine vs unvaccinated	All-cause dementia; AD	Substantial overlap with Wiemken (2022)	VHA and MarketScan estimates	Selected for primary ratio-based analysis; combined estimate used
Wiemken (2022)	USA	VHA and MarketScan; substantially overlapping periods	HZ/Tdap vaccination vs no vaccination	All-cause dementia	Substantial overlap with Scherrer (2021); same databases and author group	Multiple vaccination comparisons	Not entered with Scherrer as an independent estimate; sensitivity analysis only
Schnier (2022)	Wales, UK	SAIL Databank, 2013–2020	Mostly ZVL vs unvaccinated	All-cause dementia, AD, VaD	Likely population overlap with Eyting (2025)	Multiple dementia outcomes	All-cause estimate selected for primary ratio-based analysis
Eyting (2025)	Wales, UK	SAIL Databank; vaccination programme beginning 2013, 7-year follow-up	ZVL eligibility vs ineligibility	All-cause dementia	Likely population overlap with Schnier (2022)	Multiple RDD/IV specifications	Analysed separately as an absolute risk-difference quasi-experiment
Lophatananon (2023)	UK	CPRD Aurum/HES/ONS, 2013–31 October 2020	ZVL vs unvaccinated	All-cause dementia, AD, other dementia	Potential overlap with Douros (2023) through CPRD Aurum	Multiple dementia outcomes	All-cause estimate selected for primary analysis
Douros (2023)	UK	CPRD Gold and Aurum, 1988–2018; follow-up to 2021	HZ vaccine vs no vaccination	All-cause dementia; AD	Potential overlap with Lophatananon (2023)	Multiple vaccines and outcomes	Not entered with CPRD estimate as independent in primary analysis; sensitivity/subtype analysis only
Lophatananon (2021)	UK	UK Biobank with linked records	Zostavax vs unvaccinated	All-cause dementia	No identified overlap with other included UK datasets	Shingles and vaccination exposures	Selected for primary ratio-based analysis
Tang (2025)	USA	Optum Labs Data Warehouse, 2017–2022	One- or two-dose RZV vs unvaccinated	All-cause dementia	Potential overlap with other Optum-derived studies cannot be excluded	One- and two-dose estimates share the unvaccinated population	Complete two-dose estimate selected for primary analysis; one-dose estimate used only in exploratory dose analysis
Polisky (2025)	USA	Optum EHR, 2007–2023	RZV/ZVL vs PPSV23; RZV vs ZVL; two vs one dose	All-cause dementia	Potential overlap with Tang and Harris; linked to Schwab conference report	Shared PPSV23 comparator and multiple vaccine contrasts	Full publication retained; Schwab treated as a linked preliminary report, not an independent study or estimate.

Study/report	Country	Data source and period	Vaccine/comparator	Outcome	Potential overlap	Shared groups/multiple estimates	Handling in analyses
Harris (2023)	USA	Optum Clinformatics; 2-year look-back and 8-year follow-up	HZ vaccine vs unvaccinated	AD	Potential overlap with other Optum-derived datasets uncertain	Multiple target vaccines and matched control cohorts	Retained descriptively; not included in independent syntheses because of the AD-specific outcome and uncertain Optum overlap.
Taquet (2024)	USA	TriNetX; vaccine exposure windows 2014–2020	RZV vs ZVL; secondary influenza/Tdap comparisons	All-cause dementia	Same underlying EHR network as Taquet (2025)	Multiple comparisons share vaccine cohorts	Primary RZV-vs-ZVL RMTL estimate used for direct comparison; secondary estimates not treated as independent
Taquet (2025)	USA	TriNetX; index dates from 1 May 2023 to March 2025	AS01 shingles vaccine vs influenza vaccine	All-cause dementia	Same EHR network as Taquet (2024); individual overlap cannot be fully excluded	Shingles, RSV, combined-vaccine and influenza cohorts	RZV-vs-influenza RMTL estimate reported separately; not pooled with HR/RR/OR estimates
Pomirchy (2025)	Australia	PenCS primary-care EHR; 1 November 2016–27 March 2024	ZVL eligibility vs ineligibility	All-cause dementia	No identified overlap with other included data sources	Multiple RDD specifications	Analysed separately with quasi-experimental absolute risk-difference evidence
Schwab (2024)	USA	Optum EHR; preliminary conference report	RZV and ZVL comparisons	All-cause dementia	Linked preliminary report of Polisky (2025)	Same or substantially overlapping study framework and vaccine cohorts	Counted as a linked report but not as a separate study or independent effect estimate; the full Polisky publication was used for quantitative analyses

Notes: Potential overlap was determined from the reported database, country, study period, eligibility criteria, author group, and exposure/comparator definitions. Individual-level overlap could not be quantified because participant identifiers were unavailable. Studies using the same database were not necessarily exact duplicates but were treated as potentially correlated when populations or calendar periods could overlap. Only one estimate per study population or data-source cluster was selected for a given conventional meta-analysis. Quasi-experimental absolute risk differences and RMTL ratios were analysed separately from conventional HRs, RRs, and ORs.

Supplementary Table S4. Extracted Effect Estimates and Their Analysis-Specific Roles

Author (Year)	Comparison (Exposed vs. Control)	Vaccine Type	Dose	Effect Measure	Effect Value	95% CI Lower	95% CI Upper	Analysis Role
Primary analysis (one estimate per independent data-source cluster)								
Schnier (2022)	HZ vaccine vs. unvaccinated	Predominantly ZVL	NA	HR	0.72	0.69	0.75	Primary / conventional ZVL platform analysis
Lophatananon (2023)	HZ vaccine vs. unvaccinated (all-cause dementia)	ZVL	NA	HR	0.78	0.77	0.79	Primary / conventional ZVL platform analysis
Tang (2025)	Complete RZV vaccination vs. unvaccinated	RZV	2 doses	HR	0.68	0.67	0.70	Primary / single conventional RZV estimate / indirect dose evidence
Scherrer (2021)	HZ vaccine vs. unvaccinated (VHA and MarketScan combined)	Predominantly ZVL	NA	HR	0.69	0.66	0.71	Primary / conventional ZVL platform analysis
Lophatananon (2021)	Zostavax vs. unvaccinated	ZVL	NA	OR	0.808	0.657	0.993	Primary / conventional ZVL platform analysis
Quasi-experimental evidence (reported individually; not pooled)								
Eyting (2025)	ZVL eligibility vs. ineligibility over 7 years	ZVL	Eligibility	ITT RD (percentage points)	-1.3	-2.7	-0.2	RDD evidence / reported individually
Pomirchy (2025)	ZVL eligibility vs. ineligibility over 7.4 years	ZVL	Eligibility	ITT RD (percentage points)	-1.8	-3.3	-0.4	RDD evidence / reported individually
Direct vaccine-platform comparisons (reported individually; not pooled)								
Taquet (2024)	RZV vs. ZVL	RZV vs. ZVL	NA	RMTL ratio	0.83	0.80	0.87	Direct platform comparison / reported individually
Polisky (2025)	RZV (2+ doses) vs. ZVL (5-year)	RZV vs. ZVL	2+ doses	RR	0.82	0.69	0.98	Direct platform comparison / reported individually
Dose-regimen evidence								
Tang (2025)	One-dose RZV vs. unvaccinated	RZV	1 dose	HR	0.89	0.87	0.92	Indirect dose evidence / shared unvaccinated comparator / not pooled
Tang (2025)	Two-dose RZV vs. unvaccinated	RZV	2 doses	HR	0.68	0.67	0.70	Primary / indirect dose evidence / shared unvaccinated comparator / not pooled
Polisky (2025)	RZV (2+ doses) vs. RZV (1 dose) (5-year)	RZV	2+ vs. 1 dose	RR	0.81	0.74	0.88	Only direct dose comparison / reported individually
Active-comparator evidence (reported individually; not pooled)								
Polisky (2025)	ZVL vs. PPSV23 (5-year)	ZVL	NA	RR	0.73	0.70	0.75	Active comparator / shared PPSV23 comparator / not pooled
Polisky (2025)	RZV (2+ doses) vs. PPSV23 (5-year)	RZV	2+ doses	RR	0.83	0.75	0.92	Active comparator / shared PPSV23 comparator / not pooled
Taquet (2025)	RZV vs. influenza vaccine	RZV	NA	RMTL ratio	0.82	0.74	0.91	Active comparator / different estimand / reported individually
Age-specific descriptive evidence (correlated within study; not pooled)								
Scherrer (2021), VHA	Age 65-69 years	Predominantly ZVL	NA	HR	0.61	0.54	0.68	Age-specific descriptive estimate
Scherrer (2021), VHA	Age 70-74 years	Predominantly ZVL	NA	HR	0.83	0.76	0.91	Age-specific descriptive estimate
Scherrer (2021), VHA	Age≥75 years	Predominantly ZVL	NA	HR	0.66	0.63	0.68	Age-specific descriptive estimate
Scherrer (2021), MarketScan	Age 65-69 years	Predominantly ZVL	NA	HR	1.00	0.79	1.27	Age-specific descriptive estimate
Scherrer (2021), MarketScan	Age 70-74 years	Predominantly ZVL	NA	HR	0.65	0.52	0.81	Age-specific descriptive estimate
Scherrer (2021), MarketScan	Age≥75 years	Predominantly ZVL	NA	HR	0.59	0.50	0.69	Age-specific descriptive estimate
Polisky (2025)	Women aged 80-89 years: RZV vs. PPSV23	RZV	NA	RR	0.73	0.60	0.90	Age- and sex-specific descriptive estimate

Author (Year)	Comparison (Exposed vs. Control)	Vaccine Type	Dose	Effect Measure	Effect Value	95% CI Lower	95% CI Upper	Analysis Role
Dementia-subtype descriptive evidence (correlated or potentially overlapping; not pooled)								
Schnier (2022)	Alzheimer's disease	Predominantly ZVL	NA	HR	0.81	0.77	0.86	Subtype descriptive / correlated within study
Schnier (2022)	Vascular dementia	Predominantly ZVL	NA	HR	0.66	0.61	0.71	Subtype descriptive / correlated within study
Lophatananon (2023)	Alzheimer's disease	ZVL	NA	HR	0.91	0.89	0.92	Subtype descriptive / potential CPRD overlap
Lophatananon (2023)	Other dementia	ZVL	NA	HR	0.71	0.69	0.72	Subtype descriptive / correlated within study
Douros (2023)	Alzheimer's disease	HZ vaccine	NA	OR	1.01	0.97	1.05	Subtype descriptive / potential CPRD overlap
Polisky (2025)	Alzheimer's disease: ZVL vs. PPSV23	ZVL	NA	RR	0.51	0.45	0.58	Subtype descriptive / shared population and comparator
Polisky (2025)	Vascular dementia: ZVL vs. PPSV23	ZVL	NA	RR	0.42	0.36	0.50	Subtype descriptive / shared population and comparator
Alternative-estimate sensitivity inputs (one replacement at a time; k remained 5)								
Wiemken (2022)	HZ vaccination vs. no vaccination (VHA and MarketScan combined)	HZ vaccine (unspecified)	NA	HR	0.74	0.71	0.78	Replacement for Scherrer; not added simultaneously
Douros (2023)	HZ vaccine vs. unvaccinated	HZ vaccine	NA	OR	0.95	0.92	0.98	Replacement for Lophatananon (2023); not added simultaneously
Polisky (2025)	RZV (2+ doses) vs. PPSV23 (5-year)	RZV	2+ doses	RR	0.83	0.75	0.92	Replacement for Tang; not added simultaneously

Notes: Negative RD values indicate fewer dementia diagnoses among vaccine-eligible populations.

Direct RZV-versus-ZVL estimates were not pooled because the studies reported different effect measures and estimands.

Tang's one- and two-dose estimates shared the same unvaccinated comparator; Polisky's active-comparator estimates shared the PPSV23 comparator. These correlated estimates were displayed separately and were not treated as independent.

Age- and dementia-subtype estimates were descriptive because multiple estimates were correlated within studies or potentially overlapped across databases.

Alternative estimates replaced, rather than supplemented, the corresponding primary estimate; each replacement analysis retained five independent units.

Schwab (2024) was a linked preliminary report of Polisky (2025) and was not included as a separate study or independent effect estimate.

Abbreviations: AD, Alzheimer's disease; CI, confidence interval; HR, hazard ratio; HZ, herpes zoster; ITT, intention-to-treat; NA, not applicable; PPSV23, pneumococcal polysaccharide vaccine; RD, risk difference; RMTL, restricted mean time lost; RR, risk ratio; RZV, recombinant zoster vaccine; VaD, vascular dementia; VHA, Veterans Health Administration; ZVL, zoster vaccine live.

B. Supplementary Figures (Figure S1 to S5)

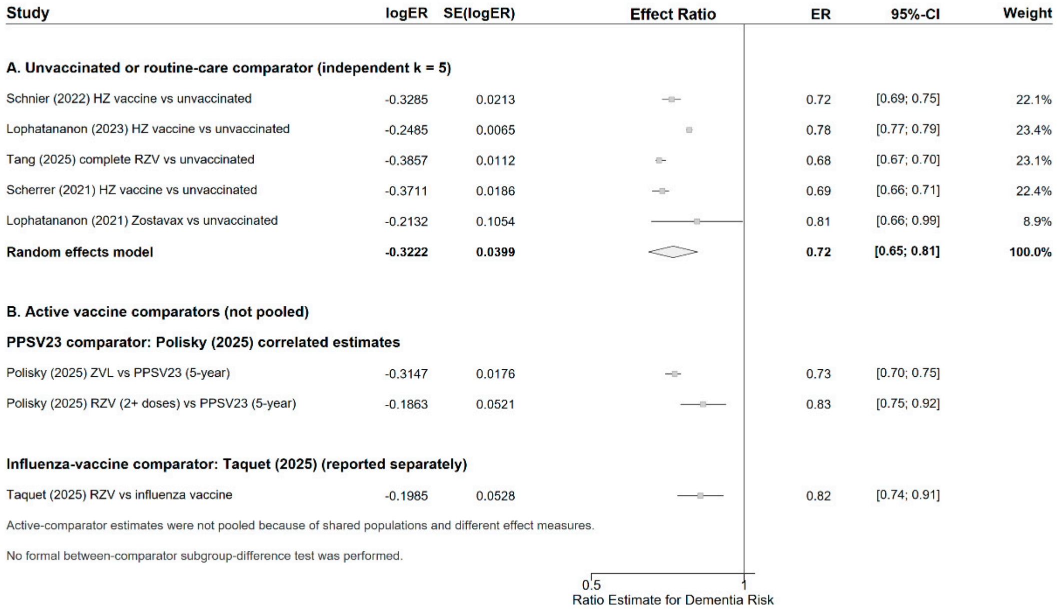

Supplementary Figure S1. Forest plot of evidence according to comparator design**.** Panel A presents the five independent comparisons using unvaccinated or routine-care controls and their random-effects summary estimate. Panel B presents active-comparator estimates from Polisky et al. using PPSV23 recipients and from Taquet et al. using influenza-vaccine recipients. The active-comparator estimates were not pooled because of shared populations and differences in effect measures. No formal between-comparator subgroup-difference test was performed. Values below 1 indicate a lower observed dementia risk among herpes zoster vaccine recipients.

Abbreviations: CI, confidence interval; ER, effect ratio; SE, standard error; PPSV23, pneumococcal polysaccharide vaccine.

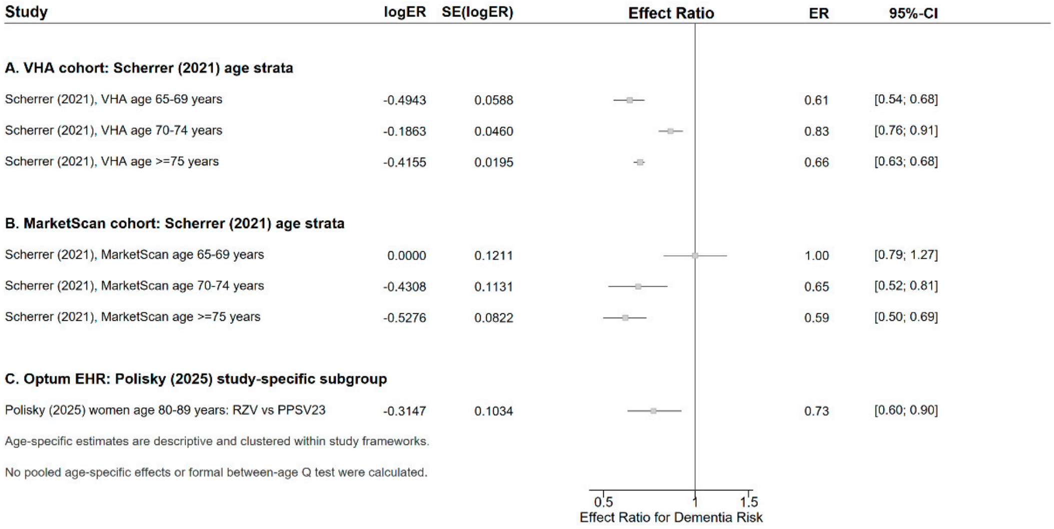

Supplementary Figure S2. Forest plot of age-specific estimates by data source. Panels A and B present age-stratified estimates from the VHA and MarketScan cohorts reported by Scherrer et al., respectively. Panel C presents the study-specific estimate among women aged 80–89 years reported by Polisky et al. Estimates were displayed descriptively because multiple age strata were clustered within the same study frameworks. No pooled age-specific effect estimates or formal between-age subgroup tests were calculated. Values below 1 indicate a lower observed dementia risk among herpes zoster vaccine recipients.

Abbreviations: HR, hazard ratio; CI, confidence interval; SE, standard error.

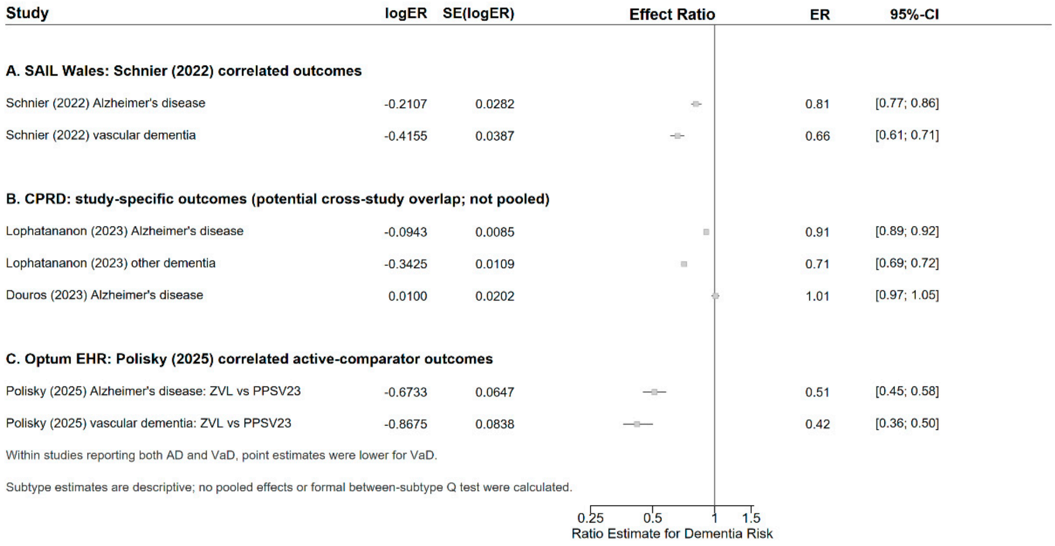

Supplementary Figure S3. Forest plot of study-specific estimates by dementia subtype. Panel A presents the correlated Alzheimer's disease and vascular dementia estimates from the SAIL Wales cohort reported by Schnier et al. Panel B presents subtype-specific estimates from CPRD studies, including potentially overlapping populations from Lophatananon et al. and Douros et al. Panel C presents the correlated active-comparator estimates for Alzheimer's disease and vascular dementia reported by Polisky et al. Within the studies reporting both Alzheimer's disease and vascular dementia, point estimates were lower for vascular dementia. No pooled subtype-specific estimates or formal between-subtype tests were calculated. Values below 1 indicate a lower observed dementia risk among herpes zoster vaccine recipients.

Abbreviations: AD, Alzheimer's disease; CI, confidence interval; ER, effect ratio; HR, hazard ratio; SE, standard error; VaD, vascular dementia.

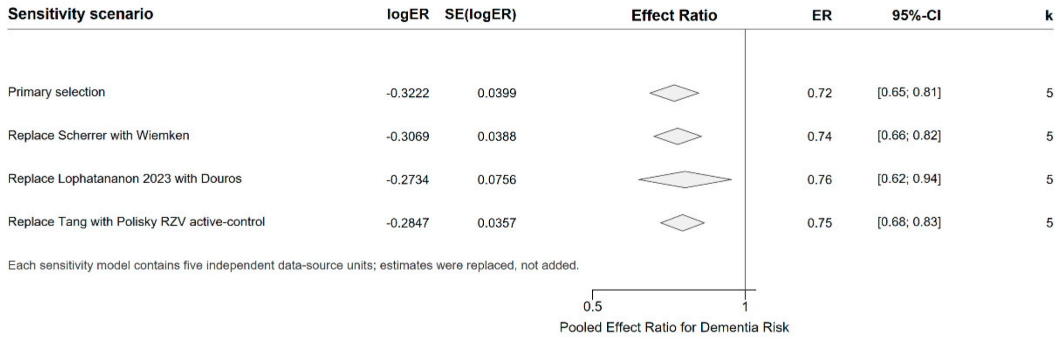

Supplementary Figure S4. Alternative-estimate sensitivity analyses retaining five independent data-source units. The primary model is shown together with three sensitivity scenarios in which Scherrer et al. was replaced by Wiemken et al., Lophatananon et al. (2023) was replaced by Douros et al., and Tang et al. was replaced by the RZV active-comparator estimate from Polisky et al. In each scenario, one estimate was replaced rather than added, and the number of independent units remained five. Diamonds represent pooled random-effects ratio estimates and 95% confidence intervals.

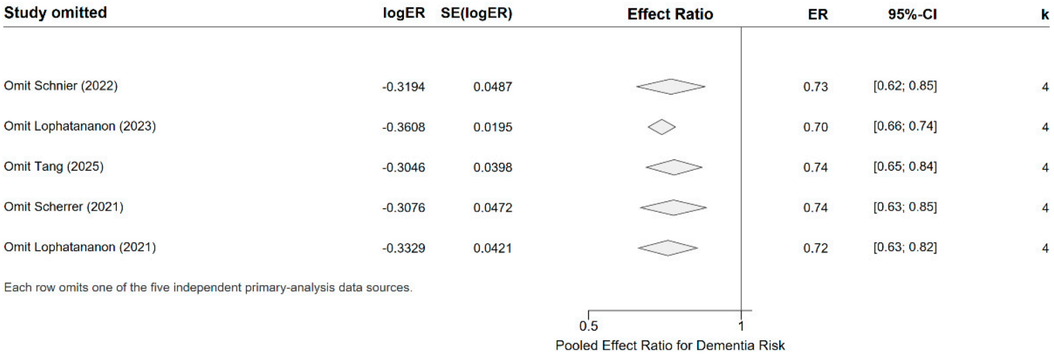

Supplementary Figure S5. Leave-one-out sensitivity analysis of the five independent primary-analysis data sources. Each row shows the pooled random-effects ratio estimate obtained after omitting one of the five independent data-source estimates included in the primary analysis. Four independent estimates remained in each iteration. Diamonds represent pooled ratio estimates and 95% confidence intervals.
